# Improving the power of clinical trials of rheumatoid arthritis by using data on continuous scales when analysing response rates: an application of the augmented binary method

**DOI:** 10.1093/rheumatology/kew263

**Published:** 2016-06-23

**Authors:** James M. S. Wason, Martin Jenkins

**Affiliations:** ^1^MRC Biostatistics Unit, Institute of Public Health, Cambridge; ^2^Global Medicines Development, AstraZeneca Pharmaceuticals, Cambridge, UK

**Keywords:** biostatistics, methodology, randomized controlled trials, rheumatoid arthritis.

## Abstract

**Objective.** In clinical trials of RA, it is common to assess effectiveness using end points based upon dichotomized continuous measures of disease activity, which classify patients as responders or non-responders. Although dichotomization generally loses statistical power, there are good clinical reasons to use these end points; for example, to allow for patients receiving rescue therapy to be assigned as non-responders. We adopt a statistical technique called the augmented binary method to make better use of the information provided by these continuous measures and account for how close patients were to being responders.

**Methods.** We adapted the augmented binary method for use in RA clinical trials. We used a previously published randomized controlled trial (Oral SyK Inhibition in Rheumatoid Arthritis-1) to assess its performance in comparison to a standard method treating patients purely as responders or non-responders. The power and error rate were investigated by sampling from this study.

**Results.** The augmented binary method reached similar conclusions to standard analysis methods but was able to estimate the difference in response rates to a higher degree of precision. Results suggested that CI widths for ACR responder end points could be reduced by at least 15%, which could equate to reducing the sample size of a study by 29% to achieve the same statistical power. For other end points, the gain was even higher. Type I error rates were not inflated.

**Conclusion.** The augmented binary method shows considerable promise for RA trials, making more efficient use of patient data whilst still reporting outcomes in terms of recognized response end points.

Rheumatology key messages

- Trials of RA treatments frequently use composite end points consisting of continuous and discrete subcomponents.
- Typical analyses of RA trials ignore information contained in the continuous subcomponent of composite end points.
- The augmented binary method better models composite end points and thereby improves power in RA trials.

## Introduction

Clinical trials in RA commonly report end points based upon assigning patients as either a responder or a non-responder to treatment according to measures of disease activity. In these so-called responder analyses, information recorded on a continuous scale is dichotomized and reduced down to a single binary outcome based upon whether patients reach a defined goal. This may be the case both for levels of improvement from baseline (e.g. ACR responder end points or HAQ-DI change from baseline) or for absolute measures of disease activity (such as DAS28, SDAI or CDAI; see Table 1). Such summaries use only the information about whether a patient’s disease activity levels are above or below a given threshold and discard information about the exact result or how far above or below this threshold the patient may lie. These analyses are therefore statistically inefficient and fail to make the maximal use of the data available, as has been well recognized in the statistical literature [1, 2], as well as in RA trials specifically [3–7].

**Table 1:** Examples of dichotomized responder end points used in clinical trials of RA

**Continuous end point**	**Responder end point**	Absolute or relative to baseline	Thresholds typically used
ACR-N	ACR20, ACR50, ACR70	Relative to baseline	20%, 50%, 70%
DAS28	Remission, low disease activity, moderate disease activity	Absolute disease activity	2.6, 3.2, 5.1
SDAI			3.3, 11, 26
CDAI			2.8, 10, 22
HAQ-DI	HAQ-DI response, according to minimally important difference	Relative to baseline	0.22, 0.25, 0.3 (various thresholds used)

SDAI: simple disease activity index; CDAI: clinical disease activity index; HAQ-DI: Health assessment questionnaire disability index.

Although most outcomes can be analysed on a continuous scale, the average change in a variable at a group level may not tell the rheumatologist how many patients have reached a meaningful level of improvement or attained a given disease activity target. This may be particularly relevant when practising a treat-to-target approach [8]. For this reason, reporting the proportion of patients achieving a minimal clinically important difference in the reduction in a patient-reported outcome, for example, may be clinically desirable in addition to purely presenting the mean change from baseline. The rheumatologist may also be more familiar with the response rates that they would expect to see for measures such as ACR20 [9], ACR50 and ACR70 rather than having an appreciation of what a desirable median ACR-N result [6] might be. Alternative outcomes, such as hybrid ACR [4], have been suggested, but have challenging distributional properties. There are therefore benefits to retaining the use of outcomes that are familiar to the rheumatology community, despite the statistical limitations of responder analyses.

Another benefit of responder analyses is that they can naturally incorporate other information into the response definition. Commonly used examples include assigning patients as non-responders if they have changes to background medications or doses, if they have rescue medications administered or if they discontinue treatment. Such an analysis answers a relevant question of how many patients can achieve meaningful clinical targets while tolerating an assigned treatment and without resorting to changes in background steroid or DMARD administration. Therefore, although withdrawal or rescue are not formally considered part of the definition of ACR20 response, typical non-responder analyses are effectively already estimating outcomes built from both continuous and binary components, as is done in the present study. This non-responder imputation for responder analyses is well established and accepted by regulators for RA trials. In contrast, the handling of missing data when working on the continuous scale is still subject to much current debate [10].

A disease area that shares similar composite end points to RA is solid tumour oncology. Patients are classed as responders if their tumour size shrinks by a prespecified level and they do not have new lesions. In that case, the augmented binary method [11] was proposed to maintain the clinically relevant responder end points but improve the precision of analyses. Compared with treating the outcome as a binary yes-or-no outcome, the augmented binary method provides a large gain in efficiency. This gain comes from the avoidance of classifying patients purely as a responder or a non-responder. Instead, it uses a suitable statistical model to account for how close patients were to being a responder.

In the present study, we propose using a similar analysis method in order to make full use of the continuous information collected from patients in clinical trials in RA while at the same time continuing to use well-recognized and understood outcomes. To illustrate the benefits of such an approach, we use the Oral SyK Inhibition in Rheumatoid Arthritis (OSKIRA-1) study [12] and consider ACR and DAS28 end points. We demonstrate that the method does not inflate the type I error rate and show that it substantially improves the power of analyses.

## Methods

### Description of the OSKIRA-1 study

The OSKIRA-1 study (NCT01197521) was a multicentre, randomized, double-blind, placebo-controlled (for 24 weeks) parallel-group study to investigate the efficacy and safety of fostamatinib in RA patients with active disease despite current treatment with MTX. The OSKIRA-1 study involved 141 centres in 17 countries. The final clinical study protocol and protocol amendments, including the final version of the informed consent form and any other written information and/or materials to be provided to the patients, was approved or given a favourable opinion in writing by an Independent Ethics Committee for each study centre.

Before enrolment of any patient into the study, the final clinical study protocol, including the final version of the informed consent form, was approved by the national regulatory authority or a notification to the national regulatory authority was done, according to local regulations. All patients provided written informed consent. Nine hundred and eighteen patients were randomized and received at last one dose of study drug. Patients were randomized (1:1:1) to receive fostamatinib 100 mg twice daily, fostamatinib 100 mg twice daily for 4 weeks and then 150 mg once daily, or placebo, on a background of MTX treatment. The trial was blinded for 52 weeks, with placebo patients switching to fostamatinib treatment at week 24 or at week 12 if requiring early rescue. In the present study, we consider only the period up to the primary end point at week 24. In the co-primary analyses, a statistically significant difference in ACR20 response rates was seen compared with placebo for both doses, but neither dose demonstrated statistical significance in radiographic scores. The results of this study have been reported previously [12] and were not deemed sufficient to seek regulatory submission in RA upon programme completion. The study is used here to illustrate the new methodology, with no intention to challenge the conclusions of the original analyses.

### RA end points

We consider the ACR and the DAS28 end points, using CRP as the acute phase reactant [13]. In all cases, the end point is classified based on data up to and including 24 weeks.

For the ACR end points, we define response according to the ACR20, ACR50 and ACR70 categorization. In each case, the patient is classed as a responder if they demonstrate a given percentage improvement in their swollen joint count, tender joint count and three of the five remaining core set measures. This is exactly equivalent to defining a patient as a responder if their ACR-N score [6] was greater than or equal to the relevant threshold (20, 50 and 70, respectively) at 24 weeks. Applying non-responder imputation, the patient must reach this threshold without having been withdrawn, had background medication changes or been given rescue therapy. As such, it is possible to model the ACR outcomes using an equivalent underlying model of ACR-N.

For the DAS28 end points, patients were similarly classified as a responder if their DAS28 score at 24 weeks was below an absolute threshold (2.6 or 3.2) and they had not been withdrawn, had background medication changes or been given rescue therapy.

### Augmented binary method

A full technical description of the notation and methodology used for the augmented binary method is given in the supplementary methods, available at *Rheumatology* Online. The augmented binary method can be used to analyse any composite outcomes that consist of a continuous component (e.g. any of the ones in Table 1). It provides an estimate of the difference in probability of a patient being a responder between two treatment arms.

For the ACR outcomes, we consider the ACR-N score at 12 weeks, the ACR-N score at 24 weeks and variables which record whether a patient was withdrawn from treatment or given rescue therapy. For the DAS28 outcomes, we consider the DAS28 score at 12 weeks, the DAS28 score at 24 weeks and the withdrawal/rescue variables. In both cases, a continuous generalized estimating equation model is fitted to the relevant score at 12 and 24 weeks, which is adjusted for treatment arm and baseline DAS28 score. A logistic regression model is fitted to model the probability of a patient being withdrawn from treatment or given rescue therapy between baseline and 12 weeks; a second logistic regression model is used to model the probability of withdrawal or rescue therapy between 12 and 24 weeks. In the former case, the treatment arm and baseline DAS28 score are included as covariates in the model; in the latter case, the treatment arm and outcome score at 12 weeks are included as covariates. Additional covariates can also be adjusted for if desirable.

The augmented binary method then combines these three models in order to estimate various quantities of interest that compare the response probabilities between arms, such as the difference or odds ratio. Importantly, it also provides CIs, allowing one to test for a significant difference between arms. In the present manuscript, we present the difference in response probabilities, but equivalent details for the odds ratio and the ratio of response probabilities are provided in supplementary Tables S1 and S2, available at *Rheumatology* Online.

### Comparison method

We compared the augmented binary method with a more standard method that treats the overall composite end point as a binary outcome. As with the augmented binary method, those patients who withdrew or received rescue medication were treated as non-responders. We fitted logistic regression models to the overall responder/non-responder indicator. To ensure that the comparison was fair, we included the baseline DAS28 score as a covariate as well as the treatment arm. The method for doing this is described further in the supplementary methods, available at *Rheumatology* Online. This is referred to henceforth as the standard binary method.

### Analyses

The augmented binary method has previously been assessed on simulated data and a small phase II cancer trial [11]. In the present analysis, we base all assessment of its performance on the OSKIRA-1 study. We first present the results of analysing the trial using standard and augmented binary methods.

Second, we sought to determine whether the augmented binary method causes any inflation in the type I error rate. We sampled a total of 5000 replicate data sets based on the OSKIRA-1 data; in each data set, the treatment assignment variables were randomly shuffled. This simulates the situation where there is no difference in the effectiveness of the two treatment arms. Each replicate data set was then analysed using both methods, and the estimated treatment effect and 95% CI were recorded. The type I error rate was estimated as the proportion of replicates in which the 95% CIs did not contain zero. Assuming the method does not cause problems with the type I error rate, this proportion should be close to 5%. From these replicates, we also recorded the width of the CIs. If the augmented binary method improves the power of RA trials, then the CI width should be narrower on average than that of the standard binary method.

A third analysis examined the power of the two methods for smaller trials. We varied the size of trial considered from 100 to 300 in increments of 10. For each size, 5000 replicates were generated. In each replicate, the specified number of patients was randomly sampled without replacement from the OSKIRA-1 data set, and both methods were applied to the resulting data set. The estimated treatment effect difference and 95% CI were recorded. The proportion of CIs that did not contain zero was used to estimate the power of both methods. In addition, we examined the correlation between the estimated treatment effect from both methods for a trial size of 200.

## Results

### Analysis of OSKIRA-1 data set

Table 2 shows the results from the original OSKIRA-1 data set. For all ACR and DAS28 categorizations, the three treatment effect summaries were found using the standard binary and augmented binary methods. The results show that for individual data sets, the two methods can give moderately different estimated treatment effects. However, the estimates for the augmented binary method remain within the bounds of the CI of the standard methods (and vice versa). In the case of ACR20, the augmented binary method gives a higher estimated difference between arms (19% compared with 13% with the standard binary method). For ACR50, ACR70 and the two DAS28 end points, the standard binary method gives higher estimated differences, but with response rates generally differing between methods by only ∼2%. In all cases, the CI from the two methods overlaps, usually substantially. There were no disagreements between the two methods in whether statistical significance was reached; thus, the conclusions of the OSKIRA-1 study would not have been affected from the use of the augmented binary method.

**Table 2:** Analysis of ACR and DAS28 end points in the OSKIRA-1 data set

	Standard binary method			Augmented binary method		
End point	**Fostamatinib 100 mg response rate**	Placebo response rate	Difference (95% CI)	**Fostamatinib 100 mg response rate**	Placebo response rate	Difference (95%CI)
ACR20	0.47 (0.42, 0.53)	0.34 (0.29, 0.39)	0.13 (0.05,0.21)	0.53 (0.49, 0.58)	0.34 (0.29, 0.39)	0.19 (0.13, 0.26)
ACR50	0.23 (0.18, 0.27)	0.08 (0.05, 0.11)	0.15 (0.09,0.20)	0.26 (0.22, 0.30)	0.13 (0.10, 0.16)	0.13 (0.08, 0.18)
ACR70	0.10 (0.06, 0.13)	0.02 (0.00, 0.04)	0.08 (0.04,0.11)	0.10 (0.09, 0.12)	0.04 (0.03, 0.05)	0.06 (0.04, 0.08)
DAS28 <3.2	0.26 (0.21, 0.30)	0.11 (0.07, 0.14)	0.15 (0.09,0.21)	0.25 (0.21, 0.28)	0.12 (0.09, 0.14)	0.13 (0.08, 0.17)
DAS28 <2.6	0.13 (0.09, 0.16)	0.05 (0.02, 0.07)	0.08 (0.04,0.12)	0.12 (0.10, 0.15)	0.05 (0.04, 0.06)	0.07 (0.05, 0.10)

The values are the response rate (i.e. proportion of patients who respond) and 95% CIs in parentheses.

Table 2 also shows that the CI width is narrower for the augmented binary method, indicating that it gives a more precise estimate of the probability of response.

### Type I error rate

Table 3 shows the results of both methods when permutation testing was used to simulate no difference between arms. In all cases, the estimated type I error rate of the augmented binary method was consistent, with the true value being 5%. In most cases, the standard binary method had the correct type I error rate; for the difference in response probabilities outcome for DAS28 <3.2, there was evidence of a small inflation. There was no evidence of inflation when the odds ratio was used, so we conclude that the inflation here is most likely to be because of the delta-method used to convert from odds ratios to the difference in response probabilities. These results indicate that there is no evidence of type I error rate inflation for the augmented binary method when realistic data are used.

**Table 3:** Type I error rate and CIs for standard and augmented binary methods

Statistical property	Difference	
	**Standard binary (%)**	**Augmented binary (%)**
ACR20		
Type I error rate (%)	5.1	5.1
Average width of CI	15.7	13.2
Relative percentage reduction in width	15.8	
ACR50		
Type I error rate	4.9	5.2
Average width of CI	11.6	9.5
Relative percentage reduction in width	17.9	
ACR70		
Type I error rate	4.6	5.0
Average width of CI	7.5	4.7
Relative percentage reduction in width	38.1	
DAS28 <3.2		
Type I error rate	5.9	5.2
Average width of CI	12.1	8.8
Relative percentage reduction in width	27.4	
DAS28 <2.6		
Type I error rate	5.0	5.3
Average width of CI	9.0	5.4
Relative percentage reduction in width	40.1	

For each outcome, 5000 simulation replicates are used (Monte-Carlo error ±0.6%). The average width of the CIs is on the logarithmic scale for the risk ratio and odds ratio outcomes.

Table 3 also shows the average width of the CIs. In all cases, the augmented binary method reduces the average CI width compared with the standard binary method. As an illustration of the gain in efficiency, on average the estimated difference in ACR20 response rate between treatments would be known to ±7.9% with the standard binary method but to ±6.6% with the augmented binary method. This 15.8% reduction in CI width is equivalent to the augmented binary method requiring a 29.1% lower sample size for the same power. The reduction in width for other end points is even higher, which means even more dramatic reductions in sample size for the same target power. It is these higher hurdle outcomes, such as ACR70 and DAS28 < 2.6, that are more typically lacking statistical power in clinical studies and thus where gains in precision could provide the most benefit.

### Subsamples of OSKIRA-1 data set

In order to examine the power of the two methods further, we examined more closely the DAS28 <2.6 end point, where the estimated treatment effect was similar between the two methods. By sampling smaller numbers of individuals repeatedly, we plotted the estimated power for different numbers of patients.

**Fig. 1:**
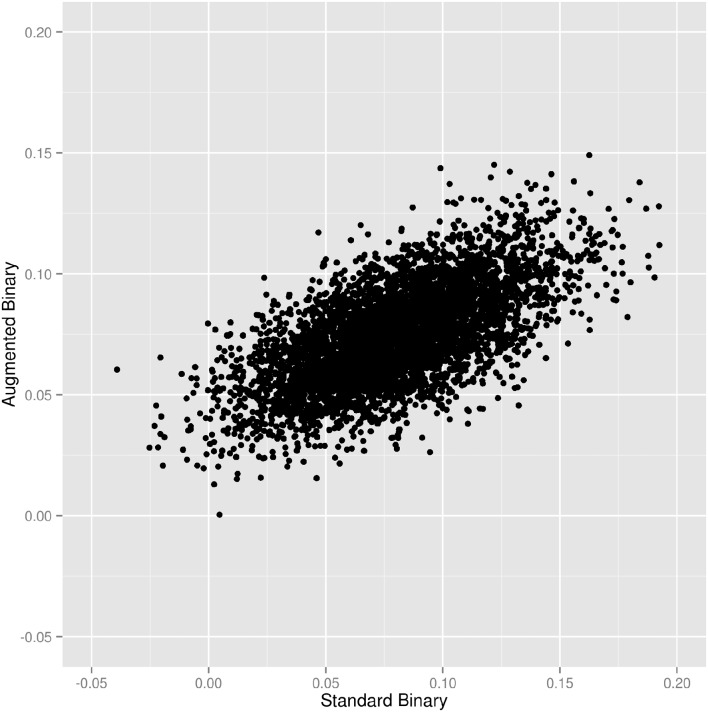
Scatter plot of estimated difference in response probability from standard and augmented binary methods Each dot represents an analysis of 200 sampled individuals from the original data set. Estimated Pearson correlation coefficient is 0.629 (95% CI: 0.612, 0.646).

For the case where 200 patients were sampled, Fig. 1 shows a scatter plot representing the estimated treatment effect (in terms of the difference in response probability between arms) from the two different methods for each replicate. It shows that there is a moderately strong correlation between estimates (Pearson correlation coefficient 0.629, 95% CI: 0.612, 0.646). This indicates that the methods reach similar conclusions but may give moderately different estimates for some individual data sets. Where the estimate given by the augmented binary method differs from that given by the standard binary method, this is more often because the augmented method is giving an estimate closer to its mean from across all the replicates. This may indicate the fact that the augmented binary method is less prone to producing chance outlying results.

**Fig. 2:**
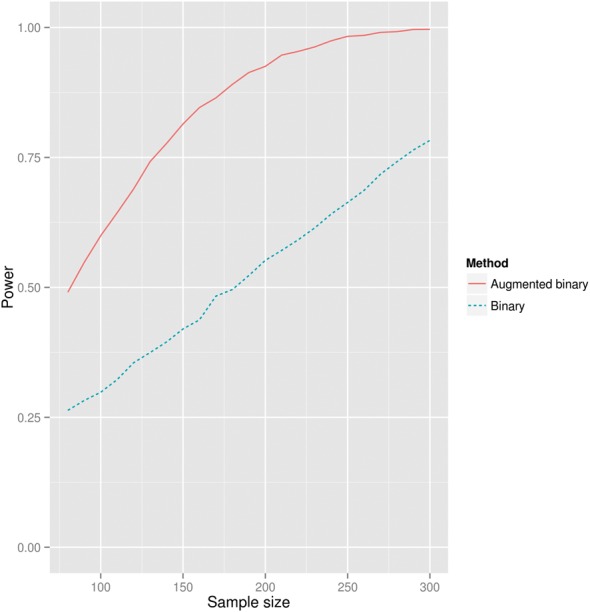
Power of standard and augmented binary methods for different sample sizes For each sample size, 5000 samples of that size from the original OSKIRA-1 data set were taken at random. The end point used is the difference in probability of achieving DAS28 <2.6 between each arm.

Figure 2 shows the power of the two methods as the sample size changes. The augmented binary method provides a large gain in power for this example data set and end point across the different sample sizes considered. As an example, if hypothesizing that the response rates observed in this study would be seen in a future study, a sample size of ∼300 would be required for 80% power at 5% two-sided type I error rate to demonstrate a significant difference with the standard binary method. To achieve the same power with the augmented binary method, ∼130 patients are required. This corresponds well to results from the type I error rate investigation, where using the augmented binary method for the DAS28 end point resulted in a large reduction in CI width.

## Discussion

We have shown that widely used binary end points in RA can be estimated considerably more precisely when the nature of the end point, being based upon the dichotomization of a continuous score, is taken into account. By using our proposed augmented binary method, the response rates for ACR and DAS28 end points could be estimated to the same degree of precision (i.e. same CI width) as the standard binary approach but using a much smaller sample size. The method does not cause any inflation to the type I error rate and can be implemented in a straightforward manner in standard statistical software packages, such as R (see https://sites.google.com/site/jmswason/supplementary-material for downloadable code). With minor changes, the methodology could be applied in a similar manner to other rheumatic diseases that use responder analyses, such as SLE, AS or PsA.

We note that in the analysis of the OSKIRA-1 data set, the augmented binary method did not change the overall conclusion (both binary and augmented binary methods showed a significant difference). In many instances, however, the improved precision may provide considerable benefits. In some cases, a trial may end with a non-definitive conclusion, such as a moderate but non-significant treatment effect. In that case, applying the augmented binary method as a prespecified secondary analysis would provide very useful additional information on whether there is a significant effect or not.

This method also retains an important advantage of responder analyses over simply testing the continuous component directly in that it allows for patients who are withdrawn from therapy or given rescue therapy to be treated as non-responders. An alternative method, proposed by Karrison *et al.* [14] in the context of phase II cancer trials, would be to test the continuous component directly but set this component to an unfavourable level for patients who failed for other reasons. However, this method does not provide such easily clinically interpretable results. Owing to this, and the fact that it produces outputs in a form that is well known and understandable to rheumatologists, we believe that our method complements existing methods by giving clinically interpretable estimates of the treatment effect for existing recognized end points whilst making best use of the data.

Although we used a relatively large study as an illustration, the augmented binary method can be applied successfully to smaller sample sizes, where gains in precision and power would be more influential. In the study by Wason and Seaman [11], the method was applied to simulated data sets with 50 patients per arm without difficulty. For smaller trials, it is possible to reduce the number of parameters in the models; for example, by modelling only one follow-up time instead of two.

As with any method based upon splitting patients above or below a threshold, questions may exist about the sensitivity of the results to the choice of this threshold. One option proposed to understand this is the use of cumulative distribution plots [15] (as often employed in RA trials [16] or to understand structural progression data). The proportion of patients with results above any threshold can be read off these plots, and we would encourage the use of such figures. However, this is largely a supportive tool, and although methods to compare distributions exist [17], these would not typically replace a more conventional analysis of response rates.

Possible extensions to the methods described here include joint modelling of individual ACR or DAS28 components rather than purely modelling the single composite index. The method could also allow for the simultaneous estimation of various outcomes using multiple thresholds based upon a single consistent model (e.g. estimating ACR20, ACR50 and ACR70 at once). Longitudinal modelling of dropouts across further time points could also be developed and could be examined under different assumptions around missing data. Such longitudinal modelling has been completed previously in RA studies [18], but without making use of the joint modelling of continuous scores and binary withdrawal factors as considered in the present manuscript. It may be possible to combine these.

Although the analyses presented here suggest that the augmented binary method could provide conventional levels of statistical power in a smaller number of patients than the existing methods, it is recommended that at this time it should be considered as a supportive analysis only and that prospective clinical trials should still be sized according to standard methods. The augmented method must be applied to different clinical data sets in order to understand the typical increases in power that it may offer. As shown in the present study, the increase in power is variable, depending on the end point, and would also depend on the response rates in the study analysed. The figures shown here should be interpreted as an illustration of the potential benefits offered and not a general rule for choosing a sample size.

### Conclusion

The augmented binary method offers a complementary analysis method for clinical trials in RA, which makes more efficient use of patient data whilst still reporting outcomes in terms of currently recognized response rates.
